# Associations among past trauma, post-displacement stressors, and mental health outcomes in Rohingya refugees in Bangladesh: A secondary cross-sectional analysis

**DOI:** 10.3389/fpubh.2022.1048649

**Published:** 2023-01-16

**Authors:** Haley Ritsema, Mari Armstrong-Hough

**Affiliations:** ^1^Department of Epidemiology, New York University School of Global Public Health, New York, NY, United States; ^2^Department of Social and Behavioral Sciences, New York University School of Global Public Health, New York, NY, United States

**Keywords:** global mental health, forced displacement, trauma, marginalized communities, refugee mental health, emergency

## Abstract

**Objective:**

The Rohingya endured intense trauma in Myanmar and continue to experience trauma related to displacement in Bangladesh. We aimed to evaluate the association of post-displacement stressors with mental health outcomes, adjusting for previously experienced trauma, in the Rohingya refugee population in Cox's Bazar, Bangladesh.

**Methods:**

We analyzed data from the Cox's Bazar Panel Survey, a cross sectional survey consisting of 5,020 household interviews and 9,386 individual interviews completed in 2019. Using logistic regression, we tested the association between post-displacement stressors such as current exposure to crime and conflict and two mental health outcomes: depression and post-traumatic stress disorder (PTSD). In adjusted analyses, we controlled for past trauma, employment status, receiving an income, food security, and access to healthcare and stratified by gender.

**Results:**

The prevalence of depressive symptoms was 30.0% (*n* = 1,357) and PTSD 4.9% (*n* = 218). Most (87.1%, *n* = 3,938) reported experiencing at least one traumatic event. Multiple post-displacement stressors, such as current exposure to crime and conflict (for men: OR = 2.23, 95% CI = 1.52–3.28, *p* < 0.001; for women: OR = 1.92, 95% CI = 1.44–2.56, *p* < 0.001), were associated with higher odds of depressive symptoms in multivariable models. Trauma (OR = 4.98, 95% CI = 2.20–11.31, *p* < 0.001) was associated with increased odds of PTSD. Living in a household that received income was associated with decreased odds of PTSD (OR = 0.74, 95% CI = 0.55–1.00, *p* = 0.05).

**Conclusion:**

Prevalence of depressive symptoms was high among Rohingya refugees living in Cox's Bazar. Adjusting for past trauma and other risk factors, exposure to post-displacement stressors was associated with increased odds of depressive symptoms. There is a need to address social determinants of health that continue to shape mental health post-displacement and increase mental healthcare access for displaced Rohingya.

## 1. Introduction

As of May 2022, 101.1 million people in the world were forcibly displaced from their homes, accounting for more than 1% of the total global population (1). People flee for reasons such as war, systematic persecution, and climate change (2). Refugees often endure severe trauma before, during, and after their flight, putting them at high risk for mental health disorders (3–8). Recent meta-analyses estimate that the prevalence of mental health disorders among refugees ranges from 22.1 to 31.5% (9, 10). This is well above the global prevalences of depression and post-traumatic stress disorder (PTSD) which are estimated at 4.4% (11) and 1.1% (12), respectively. To address this staggering mental health burden, it is critical to understand the respective contributions of past trauma related to displacement and ongoing trauma in post-displacement settings.

The Rohingya are a religious-ethnic minority group in Myanmar who have been continually displaced from their homes due to ongoing persecution by the Myanmar government. As such, they are at high risk for poor mental health outcomes related to trauma (3). For decades, Rohingya have been subjected to state-sanctioned violence such as destruction of homes and property, torture, rape and other sexual violence, murder, and massacre (3–5, 13–15). Moreover, the Myanmar government has systematically excluded the Rohingya from society by denying them their right to citizenship. This has resulted in a near total denial of their civil, political, cultural, social and economic rights (3, 16). A more detailed account of Rohingya persecution in Myanmar is further explored elsewhere in the literature (16).

Rohingya displacement was accelerated in 2017 when the Myanmar military, alongside civilian vigilante groups, committed genocide against the Rohingya with mortality estimates ranging from 6,700-9,400 Rohingya fatalities (13, 17, 18). Since, approximately 770,000 Rohingya have fled to refugee camps in Bangladesh (19). Because of this, Cox's Bazar, Bangladesh is now home to the world's largest refugee camp, housing 925,380 Rohingya as of April 2022 (19). However, Bangladesh is neither party to the 1951 United Nations Convention Relating to the Status of Refugees nor do they recognize the Rohingya as refugees *via* their own frameworks (16, 20). Therefore, Bangladesh does not recognize a legal obligation to protect or guarantee rights to Rohingya refugees.

The camps in Cox's Bazar, Bangladesh are overcrowded, unsafe and under-resourced (3, 4, 7, 21–23). An increase in the daily stressors that refugees commonly encounter in camps, such as limited work and educational opportunities, inadequate humanitarian assistance, dangerous living conditions, and limited access to healthcare, has been associated with increased symptoms of mental disorder (3, 4, 21, 24). Moreover, the daily functioning required to manage these stressors becomes increasing difficult as mental health worsens (4, 5, 25). In 2018, the majority of Rohingya in Bangladesh reported experiencing financial insecurity (95%), food insecurity (79%), lack of educational opportunities (72%), lack of appropriate living accommodations (62%), and inadequate access to clean bathrooms and sanitation facilities (62%) (3). We hypothesize that daily stressors generated by the refugee camp environment increase the burden of mental disorder in this population. This means that the environment that refugees are welcomed into may have significant implications for their ability to heal from past traumatic experiences.

Rohingya refugees have experienced an exceptional burden of severe trauma due to the systematic oppression in Myanmar and continued denial of their rights in Bangladesh (3, 13, 16). However, there is a gap in knowledge regarding the effects of post-displacement stressors on mental health outcomes and how these stressors contribute to the continuum of trauma that a Rohingya refugee experiences (7, 26). We aimed to narrow this gap by examining how post-displacement stressors might mediate, moderate or independently contribute to the effects of previously experienced trauma on mental health outcomes in the Rohingya refugee and asylum seeking population in Cox's Bazar, Bangladesh.

## 2. Materials and methods

### 2.1. Data collection

We carried out a secondary data analysis of the Cox's Bazar Panel Survey (27). The Cox's Bazar Panel Survey is a data set collected jointly by the Yale Macmillan Program on Refugees, Forced Displacement, and Humanitarian Responses, the Gender and Adolescence: Global Evidence program, and the Poverty and Equity Global Practice of the World Bank. It is a cross-sectional survey of 5,020 households, comprising 25,421 individuals and 9,386 individual adult interviews from randomly selected household adults. The household surveys collected information about the household roster, food security, consumption, assistance, assets, household income, and the anthropometrics of one randomly selected child under the age of five. The individual surveys collected information about labor market outcomes, migration history, crime and conflict, and health. Data were collected in 2019 from households in refugee camps and the host community, including refugees and Bangladeshi nationals. We excluded respondents who reported being under the age of 15 or Bangladeshi.

### 2.2. Primary outcomes

We examined two primary outcomes: depressive symptoms and PTSD. Presence of depressive symptoms was assessed *via* the Patient Health Questionnaire-9 (PHQ-9) (28), which was administered during individual adult interviews. The PHQ-9 is a nine question survey asking participants how often they have experienced common symptoms of depression over the last 2 weeks using a 4-point scale where 0 = “not at all,” 1 = “several days,” 2 = “more than half the days,” and 3 = “nearly every day.” Answers are summed for a cumulative score. A score of 10 or higher indicates that significant depressive symptoms are present and that the respondent is at clinical risk for depression. Symptoms assessed include little interest or pleasure in doing things, feeling down, depressed, or hopeless, irregular sleeping habits, feeling fatigued, poor appetite or overeating, feeling like a failure or disappointment, trouble concentrating, irregular motor movements, and thoughts of self-harm and suicide.

PTSD was assessed using the Harvard Trauma Questionnaire (HTQ) (29). The HTQ is a two-part survey tool that has been cross-culturally validated among numerous refugee populations (30, 31). However, it should be noted that the HTQ has not yet been validated among Rohingya refugees. The first part assesses exposure to traumatic events. The second part assesses clinical risk of PTSD *via* 16 questions. Participants are asked about symptoms consistent with the DSM-IV diagnostic criteria for PTSD and to what degree each symptom has bothered them. A 4-point scale is used where 1 = “not at all,” 2 = “a little,” 3 = “quite a bit,” and 4 = “extremely.” Answers are summed and divided by the number of symptoms they answered for a score. Scores of 2.5 or higher indicate significant clinical risk for PTSD. Symptoms assessed include recurrent thoughts or memories of the traumatic event(s), recurrent nightmares, feeling detached or withdrawn from people, unable to feel emotions, feeling irritable or having outbursts of anger, not wanting to interact with others outside the household, feeling as if you don't have a future, having difficulty dealing with new situations, troubled by physical problems, feeling unable to make daily plans, feeling that people do not understand what happened to you, feeling that others are hostile to you, feeling that you have no one to rely one, feeling no trust in others, feeling powerless to help others, and spending time wondering why these events happened to you. Notably, the list of symptoms assessed in this measure does not include all symptoms in the more recent DSM-5 criteria for PTSD.

### 2.3. Demographic variables and migration history

Demographic variables included if the respondent lived in the host community or a refugee camp, their country of birth, sex, age, marital status, literacy, and religion. Marital status was recoded to distinguish between being not married because of divorce, separation, or having never been married or because of being widowed (32, 33). Variables on migration history included living in the same shelter now as in July 2017, where they were living if they answered no, if they expected to return to where they were living in July 2017, and how long they expect to live where they are currently.

### 2.4. Prior trauma and current exposure to violence

Exposure to traumatic events was assessed *via* part one of the HTQ. Respondents indicated if they had experienced, witnessed, heard about it, or never experienced 12 different categories of traumatic event. Traumatic events included imprisonment, serious injury, combat situation, rape or sexual abuse, forced isolation from others, being close to death, forced separation from family members, murder of family or friends, unnatural death of family or friends, murder of stranger or strangers, being lost or kidnapped, and torture.

### 2.5. Post-displacement stressors

Post-displacement stressors included current exposure to crime and conflict, food security, employment status, receiving income from wages in the past year, monthly household income, perceived minimum monthly income needed to meet household needs, and healthcare access as measured by transit time to healthcare and healthcare costs in takas. Participant's current exposure to crime and conflict was assessed by asking if a series of different issues (bribery/corruption, harassment, theft, forced eviction, physical violence/assault, gender based violence, business disputes, family disputes, and indebtedness) were a problem in their neighborhood and if they had experienced any of them. Food security was assessed *via* an adapted version of the Household Food Insecurity Access Scale (34), a nine question scale used to determine one's experience with food insecurity based on feelings of anxiety over food access, perceptions that available food is of insufficient quantity or quality, reductions of food intake, and the consequences of those reductions. The resulting score was then coded into a binary variable that scored each respondent as either food secure or insecure.

### 2.6. Statistical analysis

We calculated frequencies and proportions for categorical variables and measures of center (mean and median) and spread (standard deviation and interquartile range) for continuous variables. We conducted bivariate tests of unadjusted associations between the covariates and depressive symptoms and PTSD, respectively, using a significance level of α = 0.05. Categorical variables were assessed *via* chi-square tests and continuous variables were assessed using *t*-tests.

Multivariable models for depressive symptoms and PTSD, respectively, were constructed using directed acyclic graphs followed by a forward-selection stepwise approach to logistic regression modeling. Starting with the variables for depressive symptoms and experiencing at least one traumatic event, covariates were added to the model one by one. All clinically significant variables (e.g., age, sex, marital status) were retained in the multivariable model regardless of statistical significance to control for potential confounding.

In both models, we tested for interaction between trauma and post-displacement stressor variables. Although not part of the initial directed acyclic graphs, bivariate analysis indicated the potential for interaction between gender, migration history, and widowhood, so these interactions were also evaluated. As appropriate, each model was stratified by gender to clarify results. All statistical analyses were completed using Stata/SE 17 Statistical Software (35).

## 3. Results

### 3.1. Descriptive statistics

Six thousand nine hundred six Rohingya individuals over the age of 15 participated in interviews (Table 1). Of these, 4,523 answered the PHQ-9, 30.0% (*n* = 1,357) of whom scored a 10 or higher on the PHQ-9, indicating a moderate to severe risk of depression. Four thousand four hundred ten respondents answered the HTQ, 4.9% (*n* = 218) of whom reported symptoms consistent with a PTSD diagnosis. Most (97.3%, *n* = 6,718) respondents reported living in a refugee camp while 2.7% (*n* = 188) reported living in the host community. The mean and median age of respondents was 31.53 and 26, respectively, indicating a right skewed distribution. 45.0% (*n* = 3,018) identified as male and 55.1% (*n* = 3,695) identified as female. 66.0% (*n* = 4,432) of respondents were married, 25.1% (*n* = 1,683) were either separated, divorced, or have never been married, and 8.9% (*n* = 597) have been widowed. Nearly all respondents described themselves as Muslim (99.9%, *n* = 6,706). Only 24.4% (*n* = 1,639) reported that they are able to read a letter, meaning the majority of respondents (75.6%, *n* = 5,071) were illiterate.

**Table 1:** Descriptive statistics of 6,906 Rohingya refugees living in Cox's Bazar, Bangladesh as it relates to trauma exposure, daily stressors, and mental health outcomes.

**Variable**	***N* (%)**
**Depression risk (PHQ-9)**	
Minimal to slight risk	3,166 (70.00)
Moderate to severe risk	1,357 (30.00)
**PTSD risk (HTQ)**	
Minimal to slight risk	4,192 (95.06)
Moderate to severe risk	218 (4.94)
**Settlement type**	
Host	188 (2.72)
Refugee camp	6,718 (97.28)
**Sex**	
Male	3,018 (44.95)
Female	3,696 (55.05)
**Age**	
Mean age (SD, range)	31.53 (15.35, 15–75)
Median age (IQR)	26 (19–40)
**Marital status**	
Married	4,432 (66.03)
Never married, divorced, or separated	1,683 (25.07)
Widowed	597 (8.89)
**Religion**	
Islam	6,706 (99.94)
Hinduism	3 (0.04)
Buddhism	8 (0.12)
**Literacy**	
No	5,071 (75.57)
Yes	1,639 (24.40)
**Living in same house/shelter before July 2017**	
No	3,996 (88.47)
Yes	521 (11.53)
**Expect to return to pre July 2017 residence**	
No	1,159 (26.74)
Yes	2,045 (47.60)
Don't know	1,025 (25.66)
**How long do you expect to live here?**	
< 1 year	88 (1.97)
One to 5 years	395 (8.83)
More than 5 years	1,213 (27.13)
Don't know	2,775 (62.07)
**Traumatic experiences**	
Any traumatic experiences listed below	
Yes	3,938 (87.09)
No	584 (12.91)
Average number of traumatic events experienced (SD, range)	3.41 (2.44, 0–10)
Median number of traumatic events experienced (IQR)	3 (1–5)
**Imprisonment**	
Experienced	646 (14.31)
Witnessed	2,150 (47.62)
Heard about it	1,441 (31.92)
No	278 (6.16)
**Serious injury**	
Experienced	1,518 (33.61)
Witnessed	1,980 (43.83)
Heard about it	706 (15.63)
No	313 (6.93)
**Combat situation**	
Experienced	2,019 (44.70)
Witnessed	1,748 (38.70)
Heard about it	627 (13.88)
No	123 (2.72)
**Rape or sexual abuse**	
Experienced	252 (5.61)
Witnessed	1,299 (28.90)
Heard about it	2,718 (60.47)
No	226 (5.03)
**Forced isolation from others**	
Experienced	1,410 (31.35)
Witnessed	1,053 (23.42)
Heard about it	1,164 (25.88)
No	870 (19.35)
**Being close to death**	
Experienced	2,435 (53.90)
Witnessed	860 (19.03)
Heard about it	744 (16.47)
No	479 (10.60)
**Forced separation from family members**	
Experienced	1,207 (26.72)
Witnessed	500 (11.07)
Heard about it	644 (14.26)
No	2,166 (47.95)
**Murder of family or friend**	
Experienced	1,540 (34.14)
Witnessed	403 (8.93)
Heard about it	466 (10.33)
No	2,102 (46.60)
**Unnatural death of family or friend**	
Experienced	1,429 (31.69)
Witnessed	528 (11.71)
Heard about it	456 (10.11)
No	2,097 (46.50)
**Murder of stranger or strangers**	
Experienced	475 (10.52)
Witnessed	1,857 (41.12)
Heard about it	1,865 (41.30)
No	319 (7.06)
**Lost or kidnapped**	
Experienced	468 (10.36)
Witnessed	993 (21.98)
Heard about it	2,390 (52.91)
No	666 (14.74)
**Torture**	
Experienced	2,018 (44.75)
Witnessed	1,116 (24.75)
Heard about it	870 (19.29)
No	505 (11.20)
**Current exposure to crime and conflict**	
Not an issue in their neighborhood, and no personal experience with it	628 (13.88)
An issue in their neighborhood, but no personal experience with it	2,139 (47.29)
An issue in their neighborhood, and personal experience with it	1,756 (38.82)
**Income met household's minimum needs in July 2017**	
Yes	3,845 (55.68)
No	3,061 (44.32)
**Currently employed?**	
Yes	979 (21.65)
No	3,543 (78.35)
**Household received income in the past year**	
Yes	1,831 (26.51)
No	5,075 (73.49)
**Household income in the last month in takas**	
Mean household income in the last month in takas (SD, range)	4,523.92 (13,009.51, 0–30,000)
Median household income in the last month in takas (IQR)	2,350 (700–5,475)
**Perceived minimum monthly income needed to meet**	
**household needs**	
Mean perceived minimum monthly income needed to meet household needs (SD, range)	10,290.33 (7,070.02, 1,000–30,000)
Median perceived minimum monthly income needed to meet household needs (IQR)	10,000 (6,000–15,000)
**Food security**	
Secure	123 (2.61)
Insecure	4,589 (97.39)
**Travel time to access healthcare in minutes**	
Mean travel time to access healthcare in minutes (SD, range)	19.48 (19.16, 2–120)
Median travel time to access healthcare in minutes (IQR)	15 (10–20)
**Cost of accessing healthcare in takas**	
Mean cost of accessing healthcare in takas (SD, range)	20.44 (63.97, 0–240)
Median cost of accessing healthcare in takas (IQR)	10 (0–20)

SD, standard deviation; IQR, interquartile range; PHQ-9, Patient Health Questionnaire-9; PTSD, post-traumatic stress disorder; HTQ, Harvard Trauma Questionnaire.

Most respondents were not yet living in Bangladesh in July 2017 (88.5%, *n* = 3,996). A minority (11.5%, *n* = 521) were already living in Bangladesh at the time of the 2017 genocide in Myanmar. 47.6% (*n* = 2,045) of respondents expected to return to where they were living prior to displacement. However, only 2.0% (*n* = 88) expected to live where they are currently for less than a year. 8.8% (*n* = 395) expected to live in Bangladesh for 1–5 years, and 27.1% (*n* = 1,213) expected to live in Bangladesh for 5 years or more. The majority of participants (62.1%, *n* = 2,775) did not know how long they expected to be displaced.

Most (87.1%, *n* = 3,938) respondents had experienced at least one traumatic event in their lifetime. The median number of experienced traumatic events was three. The three most reported traumatic events were exposure to death (53.9%, *n* = 2,435), torture (44.8%, *n* = 2,018), and combat situations (44.7%, *n* = 2,019).

#### 3.1.1. Post-displacement stressors

38.8% (*n* = 1,756) reported that crime and conflict was an issue in their neighborhood and that they had personally experienced it. 47.9% (*n* = 2,139) reported that crime was an issue in their neighborhood but they have not personally experienced it, while only 13.9% (*n* = 628) reported that there was no issue with crime and conflict in their neighborhood.

55.7% (*n* = 3,845) reported that their income prior to the 2017 genocide met their household's basic needs. When this survey was done in 2019, respondents reported receiving average and median household incomes (4,523.92 takas and 2,350 takas, respectively) that were much lower than what they reported as their average and median perceived household income needs (10,290.33 takas and 10,000 takas, respectively). 21.7% (*n* = 979) reported being currently employed and 26.5% (*n* = 1,831) reported receiving income from wages in the past year. The majority (97.4%, *n* = 4,589) of participants indicated food insecurity. Respondents reported an average transit time of 19.5 min to access healthcare services and a median time of 15 min. The average cost of healthcare reported was 20.44 takas and the median was 10 takas.

### 3.2. Unadjusted associations with depressive symptoms

Depressive symptoms (Table 2A) are associated with PTSD (odds ratio (OR) = 24.67, 95% confidence interval (CI) = 15.80–38.55, *p* < 0.001), highlighting their comorbidity in this population. Age (OR = 1.03, 95% CI = 1.03–1.04, *p* < 0.001), being female (OR = 1.41, 95% CI = 1.23–1.61, *p* < 0.001), being unmarried due to divorce, separation, or never being married (OR = 0.56, 95% CI = 0.47–0.67, *p* < 0.001), being widowed (OR = 3.08, 95% CI = 2.50–3.79, *p* < 0.001), and literacy (OR = 0.65, 95% CI = 0.55–0.76, *p* < 0.001) were associated with depressive symptoms. Living in the same shelter in July 2017 as they are now (OR = 1.32, 95% CI = 1.09–1.60, *p* = 0.005), expecting to return to where they were living in July 2017 (OR = 1.39, 95% CI = 1.17–1.63, *p* < 0.001), and expecting to live in their current shelter for 1–5 years (OR = 0.55, 95% CI = 0.34–0.89, *p* = 0.014) were all associated with depressive symptoms. Living in a refugee camp rather than the host community was not associated with depressive symptoms.

**Table 2A:** Bivariable associations with depression risk in 4,523 Rohingya refugees living in Cox's Bazar, Bangladesh.

	**Total**	**At risk for depression** ***via*** **the PHQ-9**			
**Variable**	***n***	**Yes**	**No**	***P*** **-value** ^*^	**OR (95% CI)**
**Depression risk (PHQ-9)**	–	–	–	–	–
Minimal to slight risk					
Moderate to severe risk					
**PTSD risk (HTQ)**					
Minimal to slight risk	4,192	1,112	3,080		1 (reference)
Moderate to severe risk	218	196	22	0.000	24.676 (15.797–38.545)
**Settlement type**					
Host	78	23	55		1 (reference)
Refugee camp	4,445	1,334	3,111	0.920	1.025 (0.628–1.675)
**Age**	–	–	–	0.000^†^	1.034 (1.029–1.039)
**Sex**					
Male	1,904	493	1,411		1 (reference)
Female	2,617	864	1,753	0.000	1.411 (1.237–1.608)
**Marital status**					
Married	3,211	950	2,261		1 (reference)
Never married, divorced, or separated	893	171	722	0.000	0.564 (0.469–0.677)
Widowed	415	234	181	0.000	3.077 (2.498–3.789)
**Literacy**					
No	3,477	1,113	2,364		1 (reference)
Yes	1,041	243	798	0.000	0.647 (0.551–0.759)
**Living in same house/shelter before July 2017**					
No	3,996	1,170	2,826		1 (reference)
Yes	521	184	337	0.005	1.318 (1.088–1.598)
**Expect to return to pre July 2017 residence**					
No	1,068	285	783		1 (reference)
Yes	1,901	637	1,264	0.000	1.385 (1.173–1.634)
Don't know	1,025	247	778	0.174	0.872 (0.716–1.062)
**How long do you expect to live here?**					
< 1 year	88	34	54		1 (reference)
One to 5 years	395	101	294	0.014	0.546 (0.336–0.886)
More than 5 years	1,213	357	856	0.071	0.662 (0.424–1.035)
Don't know	2,775	850	1,925	0.111	0.701 (0.453–1.085)
**Traumatic experiences**					
**Any traumatic experience**					
No	584	124	460		1 (reference)
Yes	3,938	1,233	2,705	0.000	1.691 (1.371–2.084)
**Total traumatic events**	–	–	–	0.000^†^	1.115 (1.087–1.144)
**experienced**					
**Imprisonment**					
No	278	212	66		1 (reference)
Experienced	646	210	436	0.008	1.547 (1.122–2.134)
Witnessed	2,150	707	1,443	0.002	1.574 (1.177–2.104)
Heard about it	1,441	372	1,069	0.468	1.118 (0.828–1.509)
**Serious injury**					
No	313	60	253		1 (reference)
Experienced	1,518	534	984	0.000	2.288 (1.694–3.090)
Witnessed	1,980	620	1,360	0.000	1.922 (1.428–2.587)
Heard about it	706	141	565	0.767	1.05 (0.752–1.473)
**Combat situation**					
No	123	23	100		1 (reference)
Experienced	2,019	637	1,382	0.003	2.004 (1.261–3.184)
Witnessed	1,748	547	1,201	0.004	1.980 (1.245–3.151)
Heard about it	627	149	478	0.223	1.355 (0.831–2.210)
**Rape or sexual abuse**					
No	226	49	177		1 (reference)
Experienced	252	72	180	0.084	1.445 (0.951–2.195)
Witnessed	1,299	489	810	0.000	2.181 (1.559–3.051)
Heard about it	2,718	735	1,983	0.081	1.339 (0.965–1.858)
**Forced isolation from others**					
No	870	219	651		1 (reference)
Experienced	1,410	479	931	0.000	1.529 (1.266–1.847)
Witnessed	1,053	344	709	0.000	1.442 (1.181–1.762)
Heard about it	1,164	310	854	0.458	1.079 (0.883–1.319)
**Being close to death**					
No	479	79	400		1 (reference)
Experienced	2,435	814	1,621	0.000	2.543 (1.969–3.283)
Witnessed	860	294	566	0.000	2.630 (1.989–3.478)
Heard about it	744	169	575	0.008	1.488 (1.107–2.001)
**Forced separation from family members**					
No	2,166	574	1,592		1 (reference)
Experienced	1,207	418	789	0.000	1.469 (1.262–1.711)
Witnessed	500	186	314	0.000	1.643 (1.339–2.017)
Heard about it	644	178	466	0.566	1.059 (0.870–1.290)
**Murder of family or friend**					
No	2,102	532	1,570		1 (reference)
Experienced	1,540	575	965	0.000	1.758 (1.525–2.028)
Witnessed	403	142	261	0.000	1.606 (1.280–2.014)
Heard about it	466	104	362	0.176	0.848 (0.667–1.077)
**Unnatural death of family or friend**					
No	2,097	540	1,557		1 (reference)
Experienced	1,429	546	883	0.000	1.783 (1.543–2.061)
Witnessed	528	154	374	0.112	1.187 (0.961–1.467)
Heard about it	456	112	344	0.598	0.939 (0.742–1.187)
**Murder of stranger or strangers**					
No	319	69	250		1 (reference)
Experienced	475	111	364	0.566	1.105 (0.786–1.554)
Witnessed	1,857	665	1,192	0.000	2.021 (1.523–2.682)
Heard about it	1,865	509	1,356	0.035	1.360 (1.022–1.809)
**Lost or kidnapped**					
No	666	176	490		1 (reference)
Experienced	468	168	300	0.001	1.559 (1.207–2.013)
Witnessed	993	344	649	0.000	1.476 (1.189–1.832)
Heard about it	2,390	667	1,723	0.449	1.078 (0.888–1.309)
**Torture**					
No	505	140	365		1 (reference)
Experienced	2,018	688	1,330	0.007	1.349 (1.087–1.673)
Witnessed	1,116	316	800	0.806	1.030 (0.815–1.302)
Heard about it	870	209	661	0.129	0.824 (0.642–1.058)
**Current exposure to crime and conflict**					
Not an issue in their neighborhood, and no personal experience with it	628	143	485		1 (reference)
An issue in their neighborhood, but no personal experience with it	2,139	576	1,563	0.037	1.250 (1.014–1.541)
An issue in their neighborhood, and personal experience with it	1,756	638	1,118	0.000	1.935 (1.568–2.389)
**Currently employed?**					
No	3,543	1,120	2,423		1 (reference)
Yes	979	237	742	0.000	0.691 (0.587–0.813)
**Household received income in past year**					
No	1,258	462	796		1 (reference)
Yes	3,265	895	2,370	0.000	0.651 (0.567–0.747)
**Household income in**	–	–	–	0.004†	0.999 (0.999–0.999)
**the last month in takas**					
**Perceived minimum**	–	–	–	0.279†	1.000 (0.999–1.000)
**monthly income in**					
**takas needed to**					
**meet household needs**					
**Did your income meet your household's minimum basic needs in July 2017?**					
No	2,064	679	1,385		1 (reference)
Yes	2,459	678	1,781	0.000	0.777 (0.684–0.882)
**Food security**					
Secure	106	26	80		1 (reference)
Insecure	4,417	1,331	3,086	0.215	1.327 (0.849–2.075)
**Travel time to access healthcare**	–	–	–	0.000†	1.121 (1.084–1.158)
**by 10 minute increments**					
**Cost of accessing**	–	–	–	0.000†	1.002 (1.001–1.002)
**healthcare in takas**					

^*^α = 0.05, Pearson's χ^2^ unless otherwise indicated.

^†^indicates t-test for significance.

PHQ-9, Patient Health Questionnaire-9; PTSD, post-traumatic stress disorder; HTQ, Harvard Trauma Questionnaire; OR, odds ratio; CI, confidence interval.

Experiencing at least one traumatic event (OR = 1.69, 95% CI = 1.37–2.08, *p* < 0.001) was associated with depressive symptoms. Odds of depressive symptoms increased approximately 12% with each additional traumatic event experienced (OR = 1.12, 95% CI = 1.09–1.14, *p* = 0.001). Imprisonment (OR = 1.55, 95% CI = 1.12–2.13, *p* = 0.008), serious injury (OR = 2.29, 95% CI = 1.69–3.09, *p* < 0.001), a combat situation (OR = 2.00, 95% CI = 1.26–3.18, *p* = 0.003), forced isolation from others (OR = 1.53, 95% CI = 1.27–1.85, *p* < 0.001), being close to death (OR = 2.5, 95% CI = 2.0–3.3, *p* < 0.001), forced separation from family members (OR = 1.47, 95% CI = 1.26–1.71, *p* < 0.001), murder of a family or friend (OR = 1.76, 95% CI = 1.53–2.03, *p* < 0.001), unnatural death of a family or friend (OR = 1.78, 95% CI = 1.54–2.06, *p* < 0.001), being lost or kidnapped (OR = 1.56, 95% CI = 1.21–2.01, *p* =0.001), and torture (OR = 1.35, 95% CI = 1.09–1.67, *p* = 0.007) were all associated with depressive symptoms. Witnessing the murder of strangers (OR = 2.02, 95% CI = 1.52–2.68, *p* < 0.001) or rape or sexual abuse (OR = 2.18, 95% CI = 1.56–3.05, *p* < 0.001) were also associated with depressive symptoms.

Experiencing crime and conflict in one's neighborhood (OR = 1.94, 95% CI = 1.57–2.39, *p* < 0.001) nearly doubled odds of experiencing depressive symptoms. Perceiving crime and conflict as an issue without experiencing it personally (OR = 1.25, 95% CI = 1.01–1.54, *p* = 0.037) increased odds of depressive symptoms by 25%. Other post-displacement stressors associated with depressive symptoms include being employed (OR = 0.69, 95% CI = 0.59–0.81, *p* < 0.001), receiving a household income in the past year (OR = 0.65, 95% CI = 0.59–0.75, *p* < 0.001), monthly household income (OR = 0.99, 95% CI = 0.99–0.99, *p* = 0.004), and access to healthcare as measured by transit time in 10 min increments (OR = 1.12, 95% CI = 1.08–1.16, *p* < 0.001) and cost in takas (OR = 1.002, 95% CI = 1.001–1.002, *p* < 0.001). Lastly, odds of depressive symptoms decreased for those whose 2017 income met their monthly household's perceived needs (OR = 0.77, 95% CI = 0.68–0.88, *p* < 0.001). Food security was not associated with depressive symptoms.

### 3.3. Unadjusted associations with PTSD

PTSD (Table 2B) was associated with age (OR = 1.02, 95% CI = 1.01–1.03, *p* < 0.001), being unmarried due to divorce, separation, or never being married (OR = 0.61, 95% CI = 0.40–0.94, *p* = 0.024), being widowed (OR = 2.29, 95% CI = 1.59–3.29, *p* < 0.001), and literacy (OR = 0.60, 95% CI = 0.41–0.87, *p* = 0.007). Identifying as female was not associated with PTSD. Living in the same shelter in July 2017 as now (OR = 1.47, 95% CI = 1.01–2.14, *p* = 0.047) was associated with increased odds of PTSD. Living in a refugee camp rather the host community was not associated with PTSD.

**Table 2B:** Bivariable associations with PTSD risk in 4,410 Rohingya refugees living in Cox's Bazar, Bangladesh.

	**Total**	**At risk for PTSD** ***via*** **the HTQ**			
**Variable**	***n***	**Yes (n)**	**No (n)**	***P*** **-value** ^*^	**OR (95% CI)**
**Depression risk (PHQ-9)**					
Minimal to slight risk	3,102	22	3,080	–	1 (reference)
Moderate to severe risk	1,308	196	1,112	0.000	24.676 (15.797–38.545)
**PTSD risk (HTQ)**	–	–	–	–	–
Minimal to slight risk					
Moderate to severe risk					
**Settlement type**					
Host	76	2	74	–	1 (reference)
Refugee camp	4,334	216	4,118	0.357	1.941 (0.473–7.958)
**Age**	–	–	–	0.000†	1.018 (1.009–1.027)
**Sex**					
Male	1,866	79	1,787	–	1 (reference)
Female	2,542	139	2,403	0.062	1.308 (0.986–1.736)
**Marital status**					
Married	3,144	151	2,993	–	1 (reference)
Never married, divorced, or separated	866	26	840	0.024	0.614 (0.402–0.937)
Widowed	396	41	355	0.000	2.289 (1.594–3.287)
**Literacy**					
No	3,386	184	3,202	–	1 (reference)
Yes	1,019	34	985	0.007	0.601 (0.414–0.872)
**Living in same house/shelter before July 2017**					
No	3,902	184	3,718	–	1 (reference)
Yes	502	34	468	0.047	1.468 (1.006–2.143)
**Residence before July 2017**					
Bangladesh	163	9	154	–	1 (reference)
Myanmar	3,735	174	3,561	0.611	0.836 (0.420–1.666)
Other	10	1	9	0.562	1.901 (0.217–16.694)
**Expect to return to pre July 2017 residence**					
No	1,046	59	987	–	1 (reference)
Yes	1,882	87	1,795	0.226	0.812 (0.577–1.139)
Don't know	974	38	936	0.069	0.679 (0.447–1.031)
**How long do you expect to live here?**					
< 1 year	83	16	67	–	1 (reference)
One to 5 years	393	17	376	0.000	0.189 (0.091–0.393)
More than 5 years	1,189	69	1,120	0.000	0.258 (0.142–0.469)
Don't know	2,696	116	2,580	0.000	0.188 (0.106–0.335)
**Traumatic experiences**					
**Any traumatic experience listed below**					
No	569	6	563	–	1 (reference)
Yes	3,841	212	3,629	0.000	5.482 (2.423–12.399)
**Total traumatic events experienced**	–	–	–	0.000^†^	1.329 (1.259–1.402)
**Imprisonment**					
No	260	5	255	–	1 (reference)
Experienced	620	32	588	0.036	2.776 (1.069–7.205)
Witnessed	2,124	99	2,025	0.049	2.493 (1.006–6.180)
Heard about it	1,400	82	1,318	0.013	3.173 (1.274–7.904)
**Serious injury**					
No	299	4	295	–	1 (reference)
Experienced	1,468	117	1,351	0.000	6.387 (2.339–17.440)
Witnessed	1,948	83	1,865	0.021	3.282 (1.194–9.019)
Heard about it	692	14	678	0.462	1.523 (0.497–4.665)
**Combat situation**					
No	111	0	111	–	1 (reference)
Experienced	1,956	119	1,837	0.155	1.358 (0.890–2.071)
Witnessed	1,725	71	1,654	0.644	0.900 (0.575–1.407)
Heard about it	615	28	587	–	1 (omitted because of collinearity)
**Rape or sexual abuse**					
No	211	4	207		1 (reference)
Experienced	249	10	239	0.197	2.165 (0.669–7.007)
Witnessed	1,274	87	1,187	0.010	3.793 (1.377–10.446)
Heard about it	2,658	117	2,541	0.091	2.383 (0.871–6.520)
**Forced isolation from others**					
No	845	17	828	–	1 (reference)
Experienced	1,375	132	1,243	0.000	5.172 (3.098–8.636)
Witnessed	1,041	39	1,002	0.030	1.896 (1.065–3.376)
Heard about it	1,129	30	1,099	0.354	1.330 (0.728–2.427)
**Being close to death**					
No	458	3	455	–	1 (reference)
Experienced	2,366	177	2,189	0.000	12.264 (3.900–38.561)
Witnessed	845	22	823	0.024	4.054 (1.207–13.619)
Heard about it	738	16	722	0.055	3.361 (0.974–11.599)
Forced separation from family members					
No	2,126	73	2,053	–	1 (reference)
Experienced	1,169	77	1,092	0.000	1.983 (1.428–2.754)
Witnessed	492	47	445	0.000	2.970 (2.030–4.346)
Heard about it	621	21	600	0.950	0.984 (0.601–1.613)
**Murder of family or friend**					
No	2,074	69	2,005	–	1 (reference)
Experienced	1,488	125	1,363	0.000	2.665 (1.970–3.604)
Witnessed	391	15	376	0.611	1.159 (0.656–2.048)
Heard about it	448	9	439	0.148	0.596 (0.295–1.202)
**Unnatural death of family or friend**					
No	2,066	86	1,980	–	1 (reference)
Experienced	1,380	108	1,272	0.000	1.955 (1.460–2.617)
Witnessed	507	10	497	0.023	0.463 (0.239–0.898)
Heard about it	446	14	432	0.318	0.746 (0.420–1.33)
**Murder of stranger or strangers**					
No	293	9	284	–	1 (reference)
Experienced	444	20	424	0.330	1.488 (0.668–3.316)
Witnessed	1,835	108	1,727	0.054	1.973 (0.988–3.940)
Heard about it	1,834	80	1,754	0.308	1.439 (0.714–2.899)
**Lost or kidnapped**					
No	635	36	599	–	1 (reference)
Experienced	458	41	417	0.038	1.636 (1.028–2.604)
Witnessed	977	66	911	0.382	1.205 (0.793–1.833)
Heard about it	2,335	75	2,260	0.004	0.552 (0.367–0.830)
**Torture**					
No	471	8	463	–	1 (reference)
Experienced	1,974	157	1,817	0.000	5.001 (2.440–10.250)
Witnessed	1,105	29	1,076	0.270	1.560 (0.707–3.438)
Heard about it	854	24	830	0.212	1.673 (0.746–3.755)
**Current exposure to crime and conflict**					
Not an issue in their neighborhood, and no personal experience with it	596	25	571	–	1 (reference)
An issue in their neighborhood, but no personal experience with it	2,087	88	1,999	0.981	1.005 (0.639–1.583)
An issue in their neighborhood, and personal experience with it	1,727	105	1,622	0.086	1.479 (0.947–2.311)
**Currently employed**					
No	3,452	182	3,270	–	1 (reference)
Yes	957	36	921	0.058	0.702 (0.488–1.011)
**Household received income in past year**					
No	1,211	79	1,132	–	1 (reference)
Yes	3,199	139	3,060	0.003	0.651 (0.490–0.865)
**Household income**	–	–	–	0.022†	0.999 (0.999–0.999)
**in the last month**					
**in takas**					
**Did your income meet your household's minimum basic needs in July 2017?**					
No	2,009	100	1,909	–	1 (reference)
Yes	2,401	118	2,283	0.923	0.987 (0.751–1.297)
**Food security**					
Secure	105	2	103	–	1 (reference)
Insecure	4,305	216	4,089	0.163	2.720 (0.667–11.098)
**Travel time to**	–	–	–	0.168†	1.044 (0.982–1.110)
**access healthcare by**					
**10 minute increments**					
**Cost of**	–	–	–	0.514†	1.000 (0.999–1.002)
**accessing healthcare**					
**in takas**					

^*^α = 0.05, Pearson's χ^2^ unless otherwise indicated.

^†^indicates t-test for significance.

PHQ-9, Patient Health Questionnaire-9; PTSD, post-traumatic stress disorder; HTQ, Harvard Trauma Questionnaire; OR, odds ratio; CI, confidence interval.

Experiencing at least one traumatic event (OR = 5.48, 95% CI = 2.42–12.40, *p* < 0.001) was associated with higher odds of PTSD symptoms. Odds of PTSD increased with each additional traumatic event experienced (OR = 1.33, 95% CI = 1.26–1.40, *p* < 0.001). Imprisonment (OR = 2.78, 95% CI = 1.07–7.21, *p* = 0.036), serious injury (OR = 6.39, 95% CI = 2.34–17.44, *p* < 0.001), forced isolation from others (OR = 5.17, 95% CI = 3.10–8.64, *p* < 0.001), being close to death (OR = 12.26, 95% CI = 3.90–38.56, *p* < 0.001), forced separation from family members (OR = 1.98, 95% CI = 1.43–2.75, *p* < 0.001), murder of family or friend (OR = 2.67, 95% CI = 1.97–3.60, *p* < 0.001), unnatural death of family or friend (OR = 1.96, 95% CI = 1.46–2.62, *p* < 0.001), being lost or kidnapped (OR = 1.64, 95% CI = 1.03–2.60, *p* = 0.038), and torture (OR = 5.00, 95% CI = 2.44–10.25, *p* < 0.001) were all associated with PTSD. Experiencing a combat situation or the murder of strangers were not associated with PTSD. Witnessing rape or sexual abuse (OR = 3.79, 95% CI = 1.38–10.45, *p* = 0.010) was associated with PTSD.

Crime and conflict in one's neighborhood were not associated with PTSD. Living in a household that received income from wages in the past year (OR = 0.65, 95% CI = 0.49–0.87, *p* = 0.003) was associated with reduced odds of PTSD, but there was no association between individual employment and PTSD. There was also no association between PTSD and monthly household income, having an income in July 2017 that met the household's needs, food security, or healthcare access.

### 3.4. Stratified multivariable model for depressive symptoms

No interaction effects on depressive symptoms were identified between post-displacement stressors and trauma. However, interaction effects were identified between sex and marital status, as well as sex and tenure in Bangladesh. The multivariable model for depressive symptoms is stratified based on sex (Table 3). The unstratified multivariable model for depressive symptoms with interaction terms can be found in the Supplementary material.

**Table 3:** Multivariable logistic regression model for depression among Rohingya refugees living in Cox's Bazar, Bangladesh, stratified by gender (*n* = 4,314).

	**Male (*****n*** = **1,801)**			**Female (*****n*** = **2,513)**		
**Variables**	**OR**	**95% CI**	***p*** **-value** ^*^	**OR**	**95% CI**	***p*** **-value** ^*^
**Distal exposures**						
**At least 1 traumatic event**						
No	1 (reference)	–	–	1 (reference)	–	–
Yes	1.390	0.938–2.016	0.101	1.700	1.288–2.243	0.000
**Age**	1.027	1.018–1.035	0.000	1.027	1.019–1.035	0.000
**Marital status**						
Married	1 (reference)	–	–	1 (reference)	–	–
Never married, divorced, or separated	0.784	0.552–1.113	0.174	0.779	0.597–1.016	0.065
Widowed	4.575	1.821–11.496	0.001	1.603	1.225–2.096	0.001
**Just before July 2017, were you living in this same house/shelter?**						
No	1 (reference)	–	–	1 (reference)	–	–
Yes	0.908	0.627–1.314	0.608	1.497	1.150–1.948	0.003
**Income met household needs in July 2017**						
No	1 (reference)	–	–	1 (reference)	–	–
Yes	0.776	0.620–0.973	0.028	0.706	0.589–0.845	0.000
**Proximal exposures**						
**How long you expect to live here**						
< 1 year	1 (reference)	–	–	1 (reference)	–	–
One to 5 years	0.590	0.294–1.184	0.138	0.386	0.177–0.840	0.016
More than 5 years	0.440	0.230–0.842	0.013	0.515	0.250–1.062	0.072
Don't know	0.599	0.321–1.119	0.108	0.532	0.261–1.085	0.083
**Current exposure to crime and conflict**						
No issue where they live and no personal experience with it	1 (reference)	–	–	1 (reference)	–	–
Issue where they live but no personal experience	1.198	0.811–1.769	0.365	1.290	0.973–1.709	0.076
Issue where they live and personal experience	2.229	1.515-3.280	0.000	1.922	1.444–2.559	0.000
**Currently employed**						
No	1 (reference)	–	–	1 (reference)	–	–
Yes	0.760	0.601–0.962	0.022	0.850	0.608–1.190	0.345
**Living in a household that received income from wages in past year**						
No	1 (reference)	–	–	1 (reference)	–	–
Yes	0.897	0.682–1.178	0.433	0.775	0.639–0.940	0.010
**Transit time to healthcare**	1.092	1.036–1.152	0.001	1.110	1.059–1.163	0.000
**(10 minute increments)**						

^*^α = 0.05, Pearson's χ^2^ unless otherwise indicated.

OR, odds ratio; CI, confidence interval.

Among men, odds of depressive symptoms increased with age (OR = 1.03, 95% CI = 1.02–1.04, *p* < 0.001), being widowed (OR = 4.58, 95% CI = 1.82–11.50, *p* = 0.001), and transit time to healthcare (OR = 1.09, 95% CI = 1.04–1.15, *p* = 0.001). Odds of depressive symptoms were lower among those who reported having an income that met their household's needs in July 2017 (OR = 0.78, 95% CI = 0.62–0.97, *p* = 0.028), expecting to live in their current home for more than 5 years (OR = 0.44, 95% CI = 0.23–0.84, *p* = 0.013), and being employed (OR = 0.76, 95% CI = 0.60–0.96, *p* = 0.022). Experiencing at least one traumatic event was not associated with depressive symptoms in men. Adjusting for other factors, including past exposure to trauma, personally experiencing crime and conflict in their neighborhood was associated with more than twice the odds of depressive symptoms in men (OR = 2.23, 95% CI = 1.52–3.28, *p* < 0.001).

Among women, odds of depressive symptoms increased with age (OR = 1.03, 95% CI = 1.02–1.03, *p* < 0.001), being widowed (OR = 1.60, 95% CI = 1.23–2.10, *p* = 0.001), living in the same shelter in July 2017 as they are now (OR = 1.50, 95% CI = 1.15–1.95, *p* = 0.003), and transit time to healthcare (OR = 1.11, 95% CI = 1.06–1.16, *p* < 0.001). Women who had experienced at least one traumatic event also had higher odds of depressive symptoms (OR = 1.70, 95% CI = 1.29–2.24, *p* < 0.001). Odds of depressive symptoms were lower for those whose households received income from wages in the past year (OR = 0.76, 95% CI = 0.64–0.94, *p* = 0.010). Adjusting for other factors, including past exposure to trauma, personally experiencing crime and conflict in their neighborhood was associated with nearly twice the odds of depressive symptoms in women (OR = 1.92, 95% CI = 1.44–2.56, *p* < 0.001).

### 3.5. Multivariable model for PTSD

In a multivariable model, PTSD (Table 4) was associated with experiencing at least one traumatic event (OR = 4.98, 95% CI = 2.20–11.31, *p* < 0.001), being widowed (OR = 1.71, 95% CI = 1.12–2.62, *p* = 0.013), living in the same shelter in July 2017 as now (OR = 1.50, 95% CI = 1.02–2.21, *p* = 0.041). Odds of PTSD were lower among those who expect to live in their current home more than one more year. Age and sex were not associated with PTSD. Receiving household income in the past year (OR = 0.74, 95% CI = 0.55–1.00, *p* = 0.052) was associated with decreased odds of PTSD. No interaction was found between traumatic events and current exposure to crime and conflict.

**Table 4:** Multivariable logistic regression model for PTSD among Rohingya refugees in Cox's Bazar, Bangladesh, stratified by gender (*n* = 4,349).

**Variable**	**OR**	**95% CI**	*P*-value^*^	**OR**	**95% CI**	*P*-value^*^
**Distal exposures**						
**At least 1 traumatic event**						
No	1 (reference)			1 (reference)		
Yes	5.350	2.361–12.126	0.000	4.982	2.196–11.3070	0.000
**Age**	1.009	0.998–1.020	0.101	1.008	0.997–1.019	0.149
**Sex**						
Male	1 (reference)			1 (reference)		
Female	1.225	0.904–1.659	0.191	1.217	0.894–1.656	0.212
**Marital status**						
Married	1 (reference)			1 (reference)		
Never married, divorced, or separated	0.701	0.448–1.098	0.121	0.654	0.414–1.031	0.068
Widowed	1.762	1.156–2.685	0.008	1.713	1.119–2.621	0.013
**Just before July 2017, were you living in this same house/shelter?**						
No	1 (reference)			1 (reference)		
Yes	1.485	1.013–2.177	0.043	1.498	1.016–2.209	0.041
**Proximal exposures**						
**How long you expect to live here**						
< 1 year				1 (reference)		
One to 5 years				0.174	0.083–0.366	0.000
More than 5 years				0.226	0.123–0.417	0.000
Don't know				0.182	0.101–0.327	0.000
**Received income from wages in past year**						
No				1 (reference)		
Yes				0.740	0.547–1.002	0.052

^*^α = 0.05, Pearson's χ^2^ unless otherwise indicated.

OR, odds ratio; CI, confidence interval.

## 4. Discussion

In this secondary analysis of data from 6,906 Rohingya in Cox's Bazar, Bangladesh, we found that nearly one in three adults experienced symptoms consistent with depression and nearly 5% experienced symptoms consistent with PTSD. We also found evidence that post-displacement stressors independently increased the odds of depressive symptoms among Rohingya. In particular, we found that exposure to crime and conflict in one's current neighborhood approximately doubled the odds of depressive symptoms, controlling for a wide range of other risk factors, including past trauma. This is particularly alarming as human rights organizations have reported recently that conditions for Rohingya in Bangladesh have grown increasingly violent since data were collected in 2019 (36–38). We did not find evidence that post-displacement stressors modify the effect of previously experienced trauma on mental health outcomes.

While global reported prevalence of depression is 4.4% and prevalence of PTSD is 1.1% (11, 12), 30.0 and 4.9% of respondents in this study reported symptoms consistent with depression and PTSD, respectively. Exposure to trauma is associated with depression and PTSD (39). Moreover, these estimates are consistent with the prevalence of depression in the global refugee community, estimated to range from 22.1 to 31.5% (9, 10). Indeed, our study's estimates are lower than previous studies of Rohingya displaced to Bangladesh, which range as high as 84% for depression (3) and 23.1–61% for PTSD (3, 21). This variation may be explained by differences in survey instruments, sample size, and sampling strategy. Riley et al. (3, 4) developed their own scale based upon the Hopkins Symptom Checklist in order to capture local idioms of distress. Doing so may have increased their instrument's sensitivity at the cost of its specificity. Further, previous studies were limited by smaller sample sizes (*n* = 148 and *n* = 495, respectively) (3, 4), which can reduce precision and bias estimates upwards (40). Regardless, it is clear that the burden of mental disorder among Rohingya is substantial. The need for mental health promotion and intervention among displaced Rohingya is urgent.

We also found that post-displacement stressors, such as current exposure to crime and conflict, reduced household access to income, and transit time to healthcare, significantly increase the odds of Rohingya refugees experiencing depressive symptoms. Other studies examining the impact of daily stressors on depression among Rohingya have identified stressors such as harassment by police and locals, food insecurity, income, discrimination, and sense of safety as significant predictors of negative mental health outcomes (3, 4, 40). In Bangladesh, the Rohingya have not been legally recognized as refugees by the Bangladeshi government since 1991 (20). They are not legally allowed to work, and do not have access to basic services and protections, thus exacerbating their risk for mental disorder (20, 23). These findings underscore the significant trauma that Rohingya continue to endure throughout displacement, and its poor mental health outcomes. Taken together, this work suggests that interventions to improve the post-displacement environment could significantly improve mental health outcomes.

The displacement experience affects mental health differently depending on gender. Living in Bangladesh in July 2017 – and therefore, not directly experiencing the 2017 genocide but spending more time in a Bangladeshi refugee camp - significantly *increased* women's odds of experiencing depressive symptoms. Men, on the other hand, did not have higher odds of depressive symptoms if they had lived in the camp since at least July 2017. Startlingly, this implies that, for women, protracted residence in Cox's Bazar may be worse for their mental health than surviving a genocide. Globally, refugee women are understood to experience worse mental health outcomes than men (9, 10), and Rohingya women are no exception (3, 4). Overcrowding, inadequate access to reproductive healthcare, and financial insecurity in refugee camps force women into exceedingly vulnerable positions (22, 41–43). Furthermore, displacement disrupts traditional gender norms, which can increase prevalence of gender based violence (GBV) (22, 42). Whether due to persecution in Myanmar or from dangerous conditions in Bangladesh, nearly every Rohingya woman has experienced sexual violence of some kind (15, 22, 44). Increasing access to reproductive healthcare and developing interventions that reduce GBV could improve the safety and mental health of Rohingya women.

Although harmful for both, widowhood increased odds of depressive symptoms among men more than among women. Other studies on the Rohingya have found little to no association between marital status and mental disorder (3, 4, 21, 24). However, to our knowledge, no studies specifically examine the mental health impacts of premature widowhood. For Rohingya women, marriage is a primary source of social and financial security (43), so it is surprising to see that widowhood is associated with worse outcomes among men. This could be explained, in part, by differential access to humanitarian aid. In Bangladesh, specific humanitarian programming is available for female widows, but not their male counterparts (45). However, this finding should be interpreted with caution given the small number of widowed men in our study. More research is needed on widowed refugee men as they may be an overlooked vulnerable population.

Post-displacement stressors were no longer significantly associated with PTSD in models controlling for age, sex, marital status, migration history, and past exposure to trauma. While some previous studies have identified relationships between PTSD and daily stressors (4, 21, 24), past exposure to trauma is the most important risk factor for PTSD meaning that individuals suffering from PTSD require clinical services (3–5, 21, 24, 39, 46). Although programs to address the social determinants of health are critically important, increased attention to them has come at the expense of clinical care in Bangladesh (15, 47, 48). Effective, accessible clinical care for PTSD is needed.

Notably, we found no effect of gender on likelihood of PTSD in adjusted analyses. This is not consistent with most findings on PTSD prevalence; women are up to three times as likely to suffer PTSD compared to men. There are at least two possible explanations for this surprising finding. First, the HTQ has not yet been validated among Rohingya refugee populations, so the measure of PTSD used in our analysis may be subject to bias. It is possible that the version of the HTQ used in this study is not sensitive Rohingya women's experiences of PTSD symptoms. Second, it is important to note that Rohingya in Bangladesh are not “post-trauma.” Indefinite confinement to a refugee camp without access to safety, education, and income may constitute an ongoing trauma that interferes with symptom presentation or recognition. There is great need for validation of the HTQ in a Rohingya population and for more research examining gender and trauma in long-term displacement.

### 4.1. Strengths and limitations

There are several limitations to this study. First, the data are cross-sectional. While respondents were asked to reflect on past events, there is risk of recall bias and causality cannot be established. Second, eight refugee camp blocks refused participation in data collection. Refusal was attributed to distrust of outsiders and the humanitarian community. Therefore, this study did not include a subset of the Rohingya population in Cox's Bazar that may be particularly vulnerable. Third, although the most recent version of the HTQ has been updated to reflect the full range of PTSD symptoms described in the DSM-5 (49), this study relies on the original version of the questionnaire, which was based upon the DSM-IV. As the DSM-5 criteria include several additional symptoms, it is possible that our study underreports the true prevalence of PTSD in this population. Finally, neither the PHQ-9 nor the HTQ have been formally validated as measures of depression or PTSD among Rohingya, so caution should be taken when interpreting these results. Nonetheless, both the PHQ-9 and HTQ are some of the most commonly used tools for screening for depression and PTSD, respectively, and have been validated across a diverse range of settings.

This study also has strengths. A large sample size enabled well-powered, stratified analyses. Moreover, large sample size can contribute to more accurate prevalence estimates (40). We also used a directed acyclic graph of hypothesized associations to guide the development and interpretation of multivariable models, an approach to causal diagramming that can improve inference from cross-sectional data.

### 4.2. Conclusions

Rohingya displaced to Bangladesh experience a high burden of mental disorder attributable to stressors associated with living in a refugee camp. Adjusting for other factors, including past exposure to trauma, personally experiencing crime and conflict in their present neighborhood was associated with approximately twice the odds of depressive symptoms for both men and women in Cox's Bazar. The significant impact of the refugee camp environment on mental health outcomes points to a major missed opportunity for improving refugee health. While trauma directly associated with displacement increases risk of depressive symptoms among Rohingya refugees, the quality of a refugee's immediate neighborhood environment is a more substantial—and substantially more modifiable—risk factor for depressive symptoms. Increased access to mental healthcare and the provision of safe and dignified living conditions for all Rohingya could help reduce post-displacement stress and, therefore, mitigate this mental health crisis.

Finally, the Rohingya are an understudied refugee population that deserve further inquiry. This analysis demonstrates that large data collection efforts like the CBPS are a valuable resource for public health researchers and practitioners. However, refugee camps are dynamic settings. There is a critical need for repeated collection of key data on refugee wellbeing to inform humanitarian response and public health interventions.

## Data availability statement

The original contributions presented in the study are included in the article/Supplementary material, further inquiries can be directed to the corresponding author.

## Author contributions

HR and MA-H conceptualized and designed the study. HR carried out statistical analyses in consultation with MA-H. HR drafted the initial manuscript. MA-H reviewed and revised. All authors contributed to the article and approved the submitted version.
