# Supplementary material for: Associations among past trauma, post-displacement stressors, and mental health outcomes in Rohingya refugees in Bangladesh: A secondary cross-sectional analysis
Source: Front Public Health. 2023 Jan 16;10:1048649. doi: 10.3389/fpubh.2022.1048649 (PMC9885485; doi:10.3389/fpubh.2022.1048649)
Supplement: Supplementary file 1 [file Table_1.pdf]

Supplemental Table 1: Multivariable associations with depression including interactions of 4,314 Rohingya refugees in Cox's Bazar, Bangladesh.			
Variable	OR	95% CI	P-value
**Distal Exposures**			
At least 1 traumatic event			
No	1 (reference)	--	--
Yes	1.583	1.263-1.984	0.000
Age	1.027	1.021-1.032	0.000
Sex			
Male	1 (reference)	--	--
Female	1.209	1.004-1.457	0.045
Marital status			
Married	1 (reference)	--	--
Never married, divorced, or separated	0.796	0.574-1.103	0.170
Widowed	4.325	1.734-10.788	0.002
Sex*Marital status			
Male*married	1 (reference)	--	--
Female*never married, divorced, or separated	0.990	0.663-1.477	0.959
Female*widowed	0.386	0.150-0.989	0.047
Just before July 2017, were you living in this same house/shelter?			
No	1 (reference)	--	--
Yes	0.886	0.615-1.276	0.515
Sex*Just before July 2017, were you living in this same house/shelter?			
Male*no	1 (reference)	--	--
Female*yes	1.725	1.107-2.689	0.016
Income met household needs in July 2017			
No	1 (reference)	--	--
Yes	0.731	0.636-0.842	0.000
**Proximal Exposures**			
How long you expect to live here			
Less than one year	1 (reference)	--	--
One to five years	0.477	0.284-0.799	0.005
More than five years	0.500	0.310-0.805	0.004
Don't know	0.569	0.357-0.908	0.018
Current exposure to crime & conflict			
No issue where they live and no personal experience with it	1 (reference)	--	--
Issue where they live but no personal experience	1.258	1.003-1.579	0.047

Supplemental Table 1: Multivariable associations with depression including interactions of 4,314 Rohingya refugees in Cox's Bazar, Bangladesh.			
Variable	OR	95% CI	P-value
Issue where they live and personal experience	2.044	1.626-2.570	0.000
Currently employed			
No	1 (reference	--	--
Yes	0.799	0.660-0.968	0.022
Received income from wages in past year			
No	1 (reference)	--	--
Yes	0.815	(0.696-0.953)	0.010
Transit time to healthcare (10 minute increments)	1.101	(1.063-1.140)	0.000

*α = 0.05, Pearson's χ^2 unless otherwise indicated
OR = odds ratio
CI = confidence interval
